# Eating disorders and substance use at a South African tertiary hospital over a 21-year period

**DOI:** 10.4102/sajpsychiatry.v26i0.1421

**Published:** 2020-06-24

**Authors:** Hannelie Williams, Karis Moxley, Muiruri Macharia, Martin Kidd, Gerhard P. Jordaan

**Affiliations:** 1 Department of Psychiatry, Faculty of Medicine and Health Sciences, Stellenbosch University, Cape Town, South Africa; 2 Centre for Statistical Consultation, Department of Statistics and Actuarial Sciences, Stellenbosch University, Cape Town, South Africa

**Keywords:** eating disorders, bulimia nervosa, anorexia nervosa, eating disorder not otherwise specified, substance use, South Africa

## Abstract

**Background:**

Eating disorders (EDs) and substance-related disorders pose a challenge when they co-occur and have implications for patient management. Clinical information on EDs and substance-related disorders as independent disorders is fairly well established in South Africa, but our understanding of the coexistence of these disorders is limited.

**Aim:**

To determine the prevalence, the concurrent nature and the possible trends of substance use among patients diagnosed with EDs at a South African tertiary hospital over a 21-year period.

**Setting:**

The ED unit at Tygerberg Hospital, Cape Town, South Africa.

**Methods:**

We performed a retrospective chart review of 162 patients who were treated for EDs between January 1993 and December 2014.

**Results:**

The prevalence of ED subtypes was 40.1% bulimia nervosa (BN), 33.3% EDs not otherwise specified (EDNOS) and 26.5% anorexia nervosa. Most participants (71.0%) used at least one substance. Alcohol was the most prevalent substance of choice (54.8%). Most patients had an additional psychiatric disorder (62.3%), of which major depressive disorder was the most prevalent (46.3%). Apart from the use of alcohol and cannabis, which remained consistent, the use of most other substances as well as the prevalence of BN declined during the study period.

**Conclusion:**

Understanding the prevalence and trends of EDs and the corresponding patterns of substance misuse is essential to improve service provision. This study emphasises the need to better understand the ongoing and changing behavioural trends in EDs to improve patient management.

## Introduction

The global burden of disease study 2013 has found the significant burden of eating disorders (EDs), specifically in young women living in high-income countries. However, between 1990 and 2013, the relative ranking of burden of EDs in low- and middle-income countries increased.^1^ Globally, anorexia nervosa (AN) and bulimia nervosa (BN) were responsible for 1.9 million disability adjusted life years in 2013 (DALYs).^1^ According to the US Centre on Addiction and Substance Abuse,^2^ up to 50% of individuals with an ED abuse substances, compared with 9% of the general population.

Although a substance use disorder (SUD) can occur alongside any of the ED subtypes, which include AN, BN, binge eating disorder (BED) and EDs not otherwise specified (EDNOS),^3^ the association seems to be highest among those with BN^4,5,6^ and binge/purge subtypes of AN.^7^ Nonetheless, there is a wide variability in the prevalence of SUD across ED subtypes from 2% to 6% for AN-restricting subtype, 12% to 27% for AN/binge–purge subtype and 2.9% to 48.6% for BN subtype.^6,8,9,10^

Some substances of abuse, especially alcohol, are more highly associated with EDs than others. In one clinical study, 26.9% of ED patients met the criteria for alcohol dependence.^11^ In another sample, 22.9% and 48.6% of women with BN had co-occurring alcohol dependence and alcohol abuse, respectively, compared with 8.6% in control subjects.^12^ A more recent study also found higher consumption of alcohol and other substances in patients with BN, compared with those with AN and EDNOS.^13^ Although alcohol is clearly the leading substance of choice, other substances are also frequently abused by ED patients. These include tobacco, caffeine, amphetamines, cocaine, heroin and over-the-counter medications such as diuretics or laxatives seemingly to aid weight control loss and for emotional regulation.^14^

It remains unclear as to why EDs and SUDs frequently coexist. Pearlstein suggested different models of shared and separate vulnerabilities regarding aetiology, namely addiction, genetic/familial, biological, personality/temperament and developmental models.^15^ The addiction model is based on behavioural similarities such as impulsivity, craving and denial that coexist in the presence of bio-psycho-social underpinnings.^16^ Both AN and BN, as well as SUDs, are known to be highly heritable. However, whilst these disorders share similar symptoms, evidence suggests that EDs and SUDs are distinct disorders with separate genetic transmissions.^5,17^ It is possible that bio-psycho-social etiological factors coincide and contribute in a unique way in patients with concurrent EDs and SUDs.

Although the precise mechanisms are unclear, the overall impression is that of separate vulnerabilities and also partial sharing of risk factors.

Eating disorders and SUDs pose a formidable challenge when they co-occur and have important implications for treatment and management of patients. Both disorders present with complex physical, emotional and social challenges and are independently associated with the highest mortality rates of all psychiatric disorders.^18^ Substance use may exacerbate key personality traits such as impulsivity and mood instability, which may undermine treatment for EDs.^19,20^ It can also worsen the physiological effects of ED malnutrition or starvation and impair cognition with possible consequences on efficacy of, and compliance with, medication.^21^ In addition, the risk for symptom substitution (i.e. switching from one disorder to the other) is high if treatment is targeted only towards one disorder. An integrated treatment and management strategy targeting both disorders is now recommended.^5,22^

Understanding the prevalence and trends of EDs and the corresponding patterns of substance misuse is essential to improve service provision. This is particularly important in the South African context where the advent of democracy in 1994 was characterised by rapid urbanisation and increased exposure of the South African population to Western culture in which self-worth is inordinately pegged on physical appearance,^23^ which could have potentially increased the risk for EDs in our context. The reopening of borders also saw an influx, and a growing burden, of harm associated with illicit drug use in the country.^24^

Much information concerning prevalence of EDs is available in international literature, especially for high income countries.

However, a recent review of the epidemiology of EDs on the African continent found only four studies that formally assessed ED patient populations, and information on the comorbidity of EDs and SUDs was not included.^25^ Furthermore, although clinical experience and information on EDs and substance-related disorders as independent disorders are fairly well-established in South Africa,^26,27^ there is only limited information regarding the co-existence of these disorders in our local setting. Therefore, this study aimed to retrospectively examine the prevalence, concurrent nature and possible trends of substance use (SU) among patients diagnosed with EDs at a South African tertiary hospital over a period of 21 years.

## Methods

### Study design

In 2016, we performed a retrospective chart review of patients who were treated at the ED unit at Tygerberg Hospital between January 1993 and December 2014.

### Setting

The ED unit at Tygerberg Hospital was established in 1993, which coincides with the advent of democracy in South Africa. The unit provides services for patients with EDs who are referred from primary-, secondary- and tertiary-level care, as well as from private practices. Tygerberg Hospital serves a low- to middle-income population, which is considered ‘at risk’ for mental disorders, especially owing to poverty, social adversity and high rates of unemployment in the catchment area. All patients are thoroughly evaluated by the multidisciplinary team.

### Sample

A total of 190 in- and outpatients were seen at the unit during the study period. We excluded 17 patients with ED related to medical conditions, as well as 11 patients with incomplete records (missing information relating to psychiatric diagnosis, age and gender). The final sample comprised a total of 162 patients, of which 119 were adults (≥18 years of age) and 43 were children or adolescents (10–18 years of age).

### Data collection

Patients’ age, gender, primary psychiatric diagnosis, as well as medical and/or psychiatric comorbidities were extracted by the principal investigator and subsequently collated on an anonymised Microsoft Excel spreadsheet. Other information collected included patterns of SU, as well as family history of EDs and SU. Because of the retrospective and clinically descriptive nature of this study, it was not possible to distinguish between SU and SUDs consistently. Therefore, all entries relating to SU were included. Psychiatric diagnoses were made based on criteria described in the Diagnostic and Statistical Manual of Mental Disorders^3^ at the time of assessment. In cases where there were multiple admissions during the study period, demographic and clinical variables at first assessment were reported.

### Data analysis

Continuous variables were summarised as mean ± standard deviation (SD), or median and 25th–75th percentiles and SD. Nominal variables were summarised as counts and percentages. We used appropriate parametric or non-parametric methods to test for differences between continuous variables, while Pearson’s chi-square test was used to test for differences between categorical variables. Predictors of SU in women were evaluated using logistic regression. All analyses were performed using SPSS, version 24, and statistically significant differences were established at *p* < 0.05.

### Ethical consideration

The study was approved by the Health Research Ethics Committee of Stellenbosch University (S15/09/204), as well as the Western Cape Health Research Committee. The study was granted a waiver of informed consent.

## Results

The demographic and clinical characteristics of 162 patients with EDs are summarised in Table 1.

**TABLE 1:** Demographic and clinical characteristics of patients with eating disorder (*N* = 162), showing differences between patients with and without substance use.

Variable	Overall (*N* = 162)		Substance use§				*p*
			No (*n* = 47)		Yes (*n* = 115)		
	*n*	%	*n*	%	*n*	%	
**Age mean (SD)**	26.2	9.8	23.8	9.0	27.21	10.0	0.046*
Female	155	95.7	44	27.1	111	68.5	0.409
Body mass index (SD)†	35.7	15.3	33.98	14.6	36.8	15.5	0.171
**Marital status**							
Single	119	73.5	38	23.5	81	50.0	0.318
Married	24	14.8	6	3.7	18	11.1	
Divorced/widowed	19	11.7	3	1.9	16	9.9	
**Employment status**							
Employed	56	34.6	7	4.3	49	30.2	0.001*
Unemployed	106	65.4	40	24.7	66	40.7	
**Highest level of education**‡							
Primary school (grades 1–7)	7	4.3	5	3.1	2	1.2	0.038*
High school (grades 8–11)	43	26.5	15	9.3	28	17.3	
Matric (grade 12)	79	48.8	22	13.6	57	35.2	
Tertiary qualification	29	17.9	4	2.5	25	15.4	
**Eating disorder subtype**							
Anorexia nervosa	43	26.5	14	8.6	29	17.9	0.014*
Bulimia nervosa	65	40.1	11	6.8	54	33.3	
Eating disorder NOS	54	33.3	22	13.6	32	19.8	
**Psychiatric comorbidities, yes**	101	62.3	29	17.9	74	45.7	0.914
Anxiety disorder	11	6.8	5	3.1	6	3.7	0.140
Major depressive disorder	75	46.3	18	11.1	57	35.2	0.060
Psychotic disorder	7	4.3	5	3.1	2	1.2	0.420
Personality disorder	4	2.5	1	0.6	3	1.9	0.090
OCD	4	2.5	3	1.9	1	0.6	0.050
Other¶	12	7.4	6	3.7	6	3.7	0.900
**Medical comorbidities, yes**	16	9.9	5	3.1	11	6.8	0.835
**Family history**							
Eating disorders	13	8.0	4	2.5	9	5.6	0.884
Substance use	38	23.5	11	6.8	27	16.7	0.992
Other psychiatric disorders	53	32.7	15	9.3	38	23.5	0.889

SD, standard deviation; OCD, obsessive compulsive disorder; NOS, not otherwise specified.

† , Data available for 151 patients.

‡ , Data available for only 158 patients.

§ , All percentages have been calculated relative to the total sample, *N* = 162.

¶ , Includes intellectual disability, adjustment disorder and conversion disorder, bipolar disorder.

* , Statistically significant at *p* < 0.05.

The mean age of the participants was 26.2 years (SD, 9.8; range 10–61 years). The majority of the population were female (*n* = 155, 95.7%), single (*n* = 119, 73.5%), unemployed (*n* = 106, 65.4%) and educated up to a matric level (*n* = 79, 48.8%). The overall prevalence of ED subtypes was 40.1% BN, 33.3% EDNOS and 26.5% AN.

There were no significant differences in demographic characteristics, except for the highest level of education (*p* = 0.009), between patients with different ED subtypes. Most patients had an additional psychiatric disorder (*n* = 101, 62.3%), of which major depressive disorder was the most prevalent (46.3%). Multiple comorbidities were common in this sample. Family histories included the presence of EDs (8%), SU (23.5%) and other psychiatric disorders (32.7%), such as major depressive disorder and anxiety disorders.

The majority (*n* = 115, 71.0%) of the participants used at least one substance (Table 1). There was no significant difference in age or body mass index between patients who used substances and those who did not. Substance use was most prevalent among patients with BN (*n* = 54, 33.3%). Results of the chi-square test showed that there were significant differences in employment status, education level and ED subtypes between patients who used substances and those who did not. The results of logistic regression revealed that being employed (odds ratio [OR] 3.99; confidence interval [CI]: 1.25–12.8) or having BN (OR 4.81; CI: 1.55–14.9) were significant predictors of SU in women.

Amongst patients who used at least one substance (*n* = 115, 71.0%), alcohol was the most prevalent substance of choice (54.8%), followed by laxatives (52.2%), tobacco (40.9%) and appetite suppressants (24.3%) (Table 2). The use of multiple substances was common in this sample. The use of cannabis, alcohol, laxatives, diuretics, tobacco, appetite suppressants and non-prescribed drugs was most prevalent amongst patients with BN.

**TABLE 2:** Profile of substances used by participants with different eating disorder subtypes (*n* = 115).

Substances used, *n* (%)	Overall (*n* = 115)		Eating disorder subtypes§					
			Anorexia nervosa (*n* = 29)		Bulimia nervosa (*n* = 54)		EDNOS (*n* = 32)	
	*n*	%	*n*	%	*n*	%	*n*	%
Cannabis	18	15.7	3	2.6	10	8.7	5	4.3
Alcohol	63	54.8	13	11.3	34	29.6	16	13.9
Methamphetamines	6	5.2	1	0.9	2	1.7	3	2.6
Laxatives	60	52.2	18	15.7	33	8.7	9	7.8
Diuretics	16	13.9	3	2.6	9	7.8	4	3.5
Metformin/insulin	1	0.9	0	0.0	1	0.9	0	0.0
Thyroid medications	4	3.5	0	0.0	1	0.9	0	0.0
Tobacco	47	40.9	9	7.8	23	20.0	15	13.0
Hypnotics	9	7.8	4	3.5	2	1.7	3	2.6
Appetite suppressants	28	24.3	2	1.7	21	18.3	5	4.3
Other (prescribed)†	14	14.8	7	6.1	3	2.6	7	6.1
Other (not prescribed)‡	20	17.4	3	2.6	10	8.7	7	6.1

EDNOS, eating disorders not otherwise specified.

* , Statistically significant chi-square test at *p* < 0.05.

† , Including antidepressants, benzodiazepines and mood stabilisers.

‡ , Including illicit substances, caffeine, analgesics and herbal remedies.

§ , All percentages have been calculated relative to the total sample of substance-using patients (*n* = 115).

Apart from the use of alcohol and cannabis, which remained consistent, the use of most other substances declined during the study period (Table 3). The sample was too small to assess possible statistically significant changes in the patterns of SU during the study period.

**TABLE 3:** The changing pattern of substance use amongst patients with eating disorders (*n* = 115) between 1993 and 2014.

Substance use, *n(%)*	Time period (years)§					
	1993–1999 (*n* = 46)		2000–2006 (*n* = 39)		2007–2014 (*n* = 30)	
	*n*	%	*n*	%	*n*	%
Cannabis	6	5.2	6	5.2	6	5.2
Alcohol	20	17.4	24	20.9	19	16.5
Methamphetamines	1	0.9	1	0.9	4	3.5
Laxatives	27	23.5	19	16.5	14	12.2
Diuretics	9	7.8	5	4.3	2	1.7
Metformin/insulin	1	0.9	0	0.0	0	0.0
Thyroid medications	3	2.6	0	0.0	1	0.9
Tobacco	23	20.0	11	9.6	13	11.3
Hypnotics	5	4.3	2	1.7	2	1.7
Appetite suppressants	12	10.4	13	11.3	3	2.6
Other (prescribed)†	8	7.0	5	4.3	4	3.5
Other (not prescribed)‡	4	3.5	12	10.4	4	3.5

† , Including antidepressants, benzodiazepines and mood stabilisers.

‡ , Including illicit substances, caffeine, analgesics and herbal remedies.

§ , All percentages have been calculated relative to the total sample of substance-using patients (*n* = 115).

**FIGURE 1:**
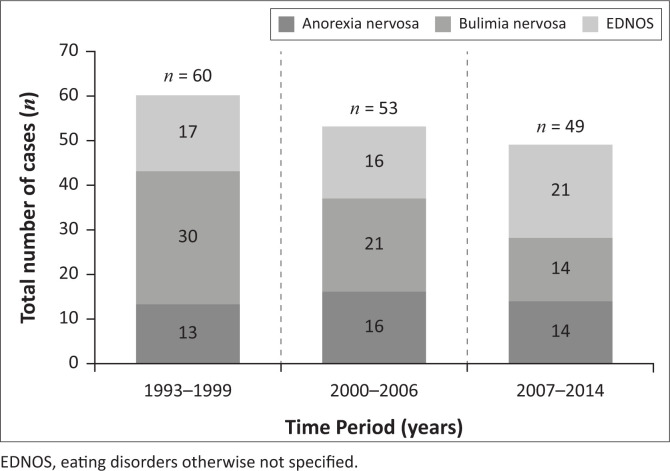
The changing patterns of cases seen at the eating disorder unit at Tygerberg Hospital between 1993 and 2014.

The prevalence of ED cases treated at the ED unit changed during the study period (Figure 1). Overall, the total number of cases treated at the ED unit at Tygerberg Hospital declined from 60 cases between 1993 and 1999 to 49 cases between 2007 and 2014. Bulimia nervosa was the most prevalent ED between 1993 and 2006, while EDNOS was most prevalent between 2007 and 2014. The number of AN cases remained constant throughout the entire study period. The sample was too small to assess possible statistically significant changes in the prevalence of different EDs during the study period.

## Discussion

This study profiled ED subtypes and concurrent SU in ED patients treated at a South African tertiary hospital between 1993 and 2014. To our knowledge, this is the first of its kind in South Africa, which we feel is interesting given that we found the rate of SU to be high. This burden of concurrent disorders may have significant clinical implications in terms of patient management and care. This report, therefore, alerts clinicians to a high index of suspicion and the need for early identification of comorbidities in South African ED populations.

Individuals with BN had the highest rates of consumption for most of the substances assessed, significantly alcohol, laxatives and appetite suppressants. Together with being employed, possibly reflecting purchasing ability, a diagnosis of BN was significantly associated with concurrent SU. Several studies have also reported higher rates of SU including amphetamines among patients with BN relative to those with AN.^7,28^

Findings between ED patients with SU and ED patients without SU are consistent with previous reports.^29^ Demographics differ in age – older patients tend to use substances more often than the younger group members. These individuals were educated with a minimum qualification of grade 12 and the most common ED being BN. This could have implications for early detection and treatment of concurrent SUDs, especially in adolescent patients and patients with BN.

This study also demonstrated the comorbidity of major depressive disorder in patients with EDs only (46.3%), and in those with EDs and SU (35.2%). Although the trend suggests that mood disorders were more prevalent in patients with EDs and SU, the difference was not statistically significant (*p* < 0.06). This association, however, requires further investigation, especially to determine the relationship between mood disorders and SU in patients with EDs (e.g. between cause and effect). Similarly, clinical awareness and early detection of mood disorders could improve outcomes in these subgroups of patients with EDs. Not having a third comparison group (SU without an ED) by the same criteria limits the final interpretation of our results.

In the 1990s, BN was the most common ED subtype in the unit but had declined twofold by 2014 relative to the other diagnoses both in proportion and absolute numbers. The number of AN patients remained relatively stable over the entire period, but rates of EDNOS almost doubled, becoming the predominant subtype by 2014.

Of every 10 patients at the unit in the 1990s, BN accounted for six, compared to two each for AN and EDNOS. However, by the 2007–2014 period, the ratio had changed to three, two and nearly five, respectively, thus showing a clear decline in relative BN rates. It is unclear whether this finding reflects trends in community ED prevalence. The pattern, however, consistent with observations that rather than a true increase in community incidence, peaks of newly diagnosed ED cases in the mid- to late 1990s may have resulted from increased awareness and detection of a relatively ‘new disorder’, which subsequently stabilised at a true level.^30^ It was suggested that increased awareness by clinicians had a greater impact on identification of BN, as it was considered a hidden illness, compared to the more recognisable AN.^30^ The proportion of patients diagnosed with AN remained relatively stable in our clinical sample and is consistent with other reports.^30,31,32^

Thus, EDNOS was not only the most frequent but also the only proportionally increasing ED in our sample supporting other studies that have identified it as the most prevalent subtype in clinical and epidemiological samples.^32,33,34^ Several factors may explain the increasing EDNOS trend in our unit. It may reflect a true rise in community prevalence in line with the recent EDNOS point prevalence estimate of 4.45%, which is five times more than the rate (0.87%) for BN.^25^ This relative increase could also have resulted from improved identification following greater awareness of symptomology not fitting AN and BN diagnoses. Furthermore, the prevalence of overweight and obesity in South Africa has increased rapidly in recent times.^36^ Weight concerns in the general population may therefor contribute to increased weight control behaviours, sub-threshold for BN and, therefore, detected as EDNOS.

Although only four patients (8.0%) indicated using methamphetamine by 2014, this was still a notable rise, compared to the previous 15 years. This rate is very consistent with the life-time prevalence of 9% reported in adolescents in Cape Town,^37^ but lower than that (37%) found in a general psychiatric inpatient population in the same hospital during 2006,^38^ and underscores the growing use of this illicit drug in this region.

The use of cannabis and alcohol remained stable throughout the study period at approximately 10% and 45%, respectively. Cigarette smoking, however, plummeted from 38% in the 1990s to 21% in 2000/2006 before rising to 27% in the last period. This paralleled trend in national smoking prevalence rates fell from 32% in 1994 to 16.4% in 2011^39^ when the government was introducing stricter tobacco control legislation and raising excise duties on cigarettes, leading to a steep rise in the cost of smoking. The use of laxatives, diuretics and appetite suppressants declined throughout the study period coinciding with the decline in the proportion of patients with bulimia who were the main consumers of these products.

This study was limited by its retrospective nature, and the information collected was constrained by the incompleteness and inaccuracy of the original data source, especially regarding SU, where reliance was solely on self-report data. As such, a distinction between abuse and dependence could not be made. Patient numbers in the three diagnostic categories were also too low to allow statistical significant comparisons over the study period. In addition, data on ethnicity were not uniformly documented (and was unusable) in spite of the importance of establishing multiethnic data for EDs^40,41^ and considering the previously common misconception that non-Caucasians are relatively unaffected by EDs.^27^

## Conclusion

This study showed that BN was the most common ED subtype in our clinic in the 1990s but has declined proportionally, compared to EDNOS at the end of the study period. Finally, the overall use of substance among patients with ED, especially BN, was high but seems to be on the decline in this study sample in recent years. It would be useful for future prospective research to assess the possible changing trends and prevalence of EDs and SU behaviour using the more recent DSM-5 criteria with a view of also distinguishing between SU and SUDs. It is anticipated that a better understanding of ongoing and changing behavioural trends in EDs would contribute to improved future patient management.
